# Engineered global regulator H-NS improves the acid tolerance of *E. coli*

**DOI:** 10.1186/s12934-018-0966-z

**Published:** 2018-07-27

**Authors:** Xianxing Gao, Xiaofeng Yang, Jiahui Li, Yan Zhang, Ping Chen, Zhanglin Lin

**Affiliations:** 1 Department of Chemical Engineering, Tsinghua University, One Tsinghua Garden Road, Beijing, 100084 China; 2 School of Biology and Biological Engineering, South China University of Technology, 382 East Outer Loop Road, University Park, Guangzhou, 510006 Guangdong China; 3 Present Address: Shenzhen Agricultural Genomics Institute, China Academy of Agricultural Sciences, 7 Pengfei Road, Dapeng District, Shenzhen, 518120 Guangdong China

**Keywords:** Acid tolerance, *E. coli*, H-NS, Global transcription machinery engineering (gTME), Error-prone PCR

## Abstract

**Background:**

Acid stress is often encountered during industrial fermentation as a result of the accumulation of acidic metabolites. Acid stress increases the intracellular acidity and can cause DNA damage and denaturation of essential enzymes, thus leading to a decrease of growth and fermentation yields. Although acid stress can be relieved by addition of a base to the medium, fermentations with acid-tolerant strains are generally considered much more efficient and cost-effective.

**Results:**

In this study, the global regulator H-NS was found to have significant influence on the acid tolerance of *E. coli*. The final OD_600_ of strains overexpressing H-NS increased by 24% compared to control, when cultured for 24 h at pH 4.5 using HCl as an acid agent. To further improve the acid tolerance, a library of H-NS was constructed by error-prone PCR and subjected to selection. Five mutants that conferred a significant growth advantage compared to the control strain were obtained. The final OD_600_ of strains harboring the five H-NS mutants was enhanced by 26–53%, and their survival rate was increased by 10- to 100-fold at pH 2.5. Further investigation showed that the improved acid tolerance of H-NS mutants coincides with the activation of multiple acid resistance mechanisms, in particular the glutamate- and glutamine-dependent acid resistance system (AR2). The improved acid tolerance of H-NS mutants was also demonstrated in media acidified by acetic acid and succinic acid, which are common acidic fermentation by-products or products.

**Conclusions:**

The results obtained in this work demonstrate that the engineering of H-NS can enhance the acid tolerance of *E. coli*. More in general, this study shows the potential of the engineering of global regulators acting as repressors, such as H-NS, as a promising method to obtain phenotypes of interest. This approach could expand the spectrum of application of global transcription machinery engineering.

**Electronic supplementary material:**

The online version of this article (10.1186/s12934-018-0966-z) contains supplementary material, which is available to authorized users.

## Background

During industrial bioprocessing microorganisms often encounter multiple stresses (e.g., high and low temperatures, extreme pH, osmotic stress and toxic compounds) [1], among which acid stress is one of the most concerned. The undissociated weak acids that accumulate during fermentation in acid-hydrolyzed lignocellulose liquor [2], or as metabolic products [3–6] and by-products [7] can permeate freely through cell membranes and then dissociate once entering into the cell cytoplasm releasing protons and conjugate bases [8–10]. The released protons increase the intracellular acidity causing DNA damage [11] and denaturation of essential enzymes [12], leading to impaired growth and fermentation yields [9]. The accumulated conjugate bases can perturb the anion balance of cells and affect their metabolism [8]. Although bases can be used to neutralize the acidic medium, thus preventing the diffusion of acids into the cells, fermentations are considered much more efficient and cost-effective with acid-tolerant strains [13–15].

Several approaches have been taken to increase the acid tolerance of bacterial strains. Mutagenesis induced by the chemical mutagen *N*-methyl-*N*′-nitro-*N*-nitrosoguanidine (NTG) has been applied to improve the acid tolerance of *Zymomonas mobilis* ZM4 [16], and adaptive laboratory evolution has been applied to improve the acid tolerance of *E. coli* [17–19]. These methods are, however, considered laborious and time-intensive since most mutations are neutral or detrimental to the desired phenotype [14, 17, 20, 21]. Other approaches, including overexpression of GadY [22] or ArrS [23], and overexpression of the arginine deaminase and glutamate decarboxylase systems by metabolic engineering [6] have also been applied. However, these approaches are not optimal since the acid tolerant phenotype of microorganisms, which includes both physiological and metabolic changes, is a complex multigenic trait [14, 24–26]. In recent years, transcriptional regulator engineering has emerged as an alternative to enhance the acid tolerance of microorganisms [1]. By this method, the expression of hundreds of genes can be perturbed simultaneously at the transcriptional level thus the tolerance can be enhanced without detailed knowledge about genotype–phenotype relationships and metabolic information [25, 27–29]. For example, the engineering of the exogenous global regulator, IrrE from *Deinococcus radiodurans*, was reported to improve the acid tolerance of *E. coli* DH5α [30]. The obtained mutant A15 had obvious growth advantage both in lysogeny broth (LB) medium supplemented with 0.05% acetate and in LB medium acidified by HCl. More recently, the endogenous global regulator CRP was engineered by error-prone PCR to improve the performance of *E. coli* DH5α at low pH [15]. The best mutant AcM1 showed almost double the growth rate as that of the control (0.113 and 0.062 h^−1^, respectively) at pH 4.24. Besides, the acid tolerance of *E. coli* DH5α was also improved by engineering the global regulator Sigma D factor (RpoD) by a random insertional–deletional strand exchange mutagenesis (RAISE) method [14]. The identified best strain Mutant VII exhibited a growth rate of 0.22 h^−1^ compared with 0.15 h^−1^ of the control.

In this work, we focused on the engineering of the histone-like nucleoid structuring factor (H-NS) to improve the acid tolerance of *E. coli*. H-NS, an abundant protein of 15.5 kDa, is one of the seven global regulators (i.e. CRP, IHF, FNR, FIS, ArcA, Lrp and H-NS) [31] and it is able to influence the expression of 5% of the genes in *E. coli* [32]. H-NS can preferentially silence the expression of horizontally transferred genes, which plays an important role in acquiring competitive fitness at minimal cost [33–35]. It can also interact with the transcription initiation and elongation complex, thereby exerting a dramatic negative effect on gene expression due to its ability to bind the AT-rich region commonly found in promoters [36–38]. H-NS regulates many genes involved in the response to environmental changes [39–41], and in particular the genes related to acid tolerance [32]. It has been reported that the knockout of *hns* can improve the survival of *E. coli* under extreme acid stress and increases the transcription of RpoS [42], which is one of the key regulators of the acid stress response [43, 44]. Further study showed that H-NS can influence the stability of RpoS by inhibiting the expression of IraD and IraM, which are anti-adaptor proteins that stabilize RpoS [45–47]. H-NS can repress almost all the structural genes and regulators involved in glutamate- and glutamine-dependent acid resistance (AR2), arginine-dependent acid resistance (AR3) and lysine-dependent acid resistance (AR4) systems [41, 48]. Besides, H-NS can repress the expression of the chaperone HdeA/B [49, 50] which are involved in the acid tolerance response of *E. coli* [26]. Taken together, these findings suggest that the engineering of H-NS might improve the acid tolerance of *E. coli*.

In this study, we first confirmed the link between H-NS and acid tolerance by investigating the effect of the knockout and overexpression of *hns* on cell growth in media acidified by HCl. Subsequently, a random mutagenesis library of *hns* was constructed by error-prone PCR and subjected to selection under acid stress. To shed some light on the mechanism behind the increase of acid tolerance, the transcriptome analysis of the strains showing the highest acid tolerance was performed. Glutamate decarboxylase (GAD) activity and the release of γ-amino butyric acid (GABA) and ammonia were further measured to test if the AR2 system was implicated. The acid tolerance of the engineered mutants was also evaluated in media supplemented with acetic or succinic acid, which are common acidic fermentation by-products or products.

## Methods

### Materials

Restriction enzymes, Q5 DNA polymerase and T4 DNA ligase were purchased from New England Biolabs (Beverly, MA, USA) and LA Taq DNA polymerase was purchased from TaKaRa Biotechnology (Dalian, China). Oligonucleotides were synthesized by Taihe Biotechnology Co., Ltd. (Beijing, China). Sequence analysis was performed either by Invitrogen (Carlsbad, CA, USA) or by Taihe Biotechnology Co., Ltd. (Beijing, China). The kits for DNA purification, gel recovery and plasmid mini-prep were purchased from Tiangen (Beijing, China).

### Strains and plasmids

The strains and plasmids used in this study are listed in Table 1. The *hns* knockout strain *E. coli* MG1655 ∆*hns* was constructed by a scarless method based on the CRISPR-Cas9 system employing pCas and pTargetT-∆*hns* [51]. To construct plasmid pBR322-*hns* (WT), the open reading frame (ORF) of *hns* gene (414 bp) and the native promoter (604 bp) were amplified together from the genome of *E. coli* MG1655 with primers *Aat*II-hns-for (CCCGGACGTCCAGCCACAGGCCCTCAATG) and *Xho*I-hns-rev (CCGCTCGAGTATTGCTTGATCAGGAAATCGTC) and inserted into plasmid pBR322 followed by the insertion of the rrnBT terminator from plasmid pEAS-1b [52].

**Table 1:** Strains and plasmids used in this study

Strains/plasmids	Genotype or description	Source
Strains		
MG	Wild type *E. coli* MG1655 strain	Lab collection
Δ*hns*	*E. coli* MG165*5* Δ*hns*	This study
H-NS(WT)	*E. coli* MG1655 pBR322-*hns* (WT), Amp^R^	This study
3-36	*E. coli* MG1655 pBR322-*hns* (3-36), Amp^R^	This study
5-30	*E. coli* MG1655 pBR322-*hns* (5-30), Amp^R^	This study
9-1	*E. coli* MG1655 pBR322-*hns* (9-1), Amp^R^	This study
9-36	*E. coli* MG1655 pBR322-*hns* (9-36), Amp^R^	This study
10-21	*E. coli* MG1655 pBR322-*hns* (10-21), Amp^R^	This study
Plasmids		
pTargetF-*cadA*	*pMB1*, sgRNA-*cadA*, Spe^R^	Gift of professor Sheng Yang
pCas	*repA101* (Ts), Pcas-*cas9*, ParaB-Red, lacI^q^, Ptrc-sgRNA-*pMB1*, Kan^R^	Gift of professor Sheng Yang
pTargetT-*Δhns*	*pMB1*, sgRNA-*hns*, Homo X1-Homo X2 (*hns*), Spe^R^	This study
pBR322	*pMB1*, rop, Amp^R^	New England Biolabs
pBR322-*hns* (WT)	*pMB1*, rop, pNat-*hns* (WT)-rrnBT, Amp^R^	This study
pBR322-*hns* (3-36)	*pMB1*, rop, pNat-*hns* (3-36)-rrnBT, Amp^R^	This study
pBR322-*hns* (5-30)	*pMB1*, rop, pNat-*hns* (5-30)-rrnBT, Amp^R^	This study
pBR322-*hns* (9-1)	*pMB1*, rop, pNat-*hns* (9-1)-rrnBT, Amp^R^	This study
pBR322-*hns* (9-36)	*pMB1*, rop, pNat-*hns* (9-36)-rrnBT, Amp^R^	This study
pBR322-*hns* (10-21)	*pMB1*, rop, pNat-*hns* (10-21)-rrnBT, Amp^R^	This study

### Library construction and selection

The random mutagenesis of *hns* was performed by standard error-prone PCR [53]. The ORF of *hns* and the native promoter were amplified from plasmid pBR322-*hns* (WT) with primers *Aat*II-hns-for and *Xho*I-hns-rev. After purification and digestion by *Aat*II and *Xho*I, the error-prone PCR product was inserted into plasmid pBR322-*hns* (WT) to replace the wild type *hns* gene and the native promoter. The ligation product was then transformed into *E. coli* MG1655 competent cells by electroporation. Cells were incubated overnight at 37 °C on LB plates containing 50 μg/mL ampicillin, and were then scraped off to create a liquid library. The library size was 10^6^ with a mutation rate controlled at 5 mutations/gene by adjusting the concentration of Mn^2+^.

The mutants were selected considering growth under moderate acid stress (pH 4.5) and survival under extreme acid stress (pH 2.5). For each round, 200 μL aliquots from 10 cultures of the library with initial OD_600_ 0.05 were separately inoculated into 10 mL LBG medium (LB medium supplemented with 2% glucose) acidified by HCl to pH 4.5 and cultivated at 37 °C, 250 rpm for 16 h. After reaching stationary phase, the cultures were diluted to OD_600_ 0.05 with fresh LBG medium of pH 4.5 and cultured to OD_600_ 0.5–0.6, then were incubated for 1 h in LBG medium acidified by HCl to pH 2.5 and then were plated on LB plates containing 50 μg/mL ampicillin. The survived cells were scraped off for the next round. This procedure was repeated for 6 rounds, then 500 clones were selected to monitor the growth curves by an automated turbidimeter (Bioscreen C, Oy Growth Curves Ab Ltd., Helsinki, Finland). The plasmids harboring H-NS mutants showing the highest acid tolerance were extracted and re-transformed into fresh *E. coli* MG1655 competent cells for confirmation.

### Growth assay

MG, Δ*hns* and strains harboring pBR322-derived plasmids were grown overnight (about 16 h) in LBG medium of pH 7.0 at 37 °C. The cultures were then diluted to initial OD_600_ 0.05 in 300 μL LBG medium of pH 7.0, LBG medium acidified by acetic acid to pH 5.35 or LBG medium acidified by HCl or succinic acid to pH 4.5. Then the cultures were incubated at 37 °C in 100-well Honeycomb microplates using an automated turbidimeter (Bioscreen C, Oy Growth Curves Ab Ltd., Helsinki, Finland) for online monitoring of OD_600_ for 24 h.

### Acid shock assay

MG, ∆*hns* and strains harboring pBR322-derived plasmids were grown overnight in LBG medium of pH 7.0 at 37 °C. The cultures were then diluted to initial OD_600_ 0.05 in LBG medium acidified by HCl to pH 4.5 and grown at 37 °C to exponential phase with OD_600_ 0.5–0.6. The cultures were diluted with fresh LBG medium of pH 4.5 to OD_600_ 0.5, then diluted 1:10 with LBG medium acidified by HCl to pH 2.5 in 96-well microplates and incubated for 1 h at 37 °C. The cultures before and after acid shock were serially diluted, plated on LB plates, incubated at 37 °C overnight and then photographed.

### GAD activity assay

Cells grown in LBG medium acidified by HCl to initial pH 4.5 were harvested in exponential phase with OD_600_ 0.5–0.6 and in stationary phase after being cultured for 16 h, by centrifugation. The pellet was washed once with phosphate buffer (20 mM, pH 7.4), pelleted again, and resuspended to 10 OD_600_/mL with potassium phosphate buffer (20 mM, pH 7.4). After sonication, the cell debris was removed by centrifugation, and the crude extracts were used for GAD activity assay. The colorimetric assay for measuring GAD activity was performed in a 96-well microplate format as previously described [54]. The standard reaction medium in each well consisted of 200 μL acetate buffer (20 mM, pH 4.8) containing 70 μM bromocresol green (BCG), 10 mM pyridoxal-5′-phosphate (PLP), 25 mM l-glutamate and 20 μL of crude extract. Blank medium without l-glutamate was used as background. The absorbance at 620 nm was monitored at 37 °C and data were collected every 1 min for 1 h using an Infinite M200 Pro plate reader (Tecan Group Ltd., Männedorf, Switzerland).

### GABA measurement

Cells grown in LBG medium acidified by HCl to initial pH 4.5 were harvested in exponential phase with OD_600_ 0.5–0.6 and in stationary phase after being cultured for 16 h, by centrifugation. The pellet was washed once with citrate buffer (25 mM, pH 4.5), pelleted again, and resuspended to 4 OD_600_/mL with 1 mL citrate buffer (25 mM, pH 4.5) supplemented with 10 mM l-glutamate. After 1 h of incubation, the culture was centrifuged and the supernatant was filtered for the analysis of GABA concentration. The concentration of GABA was measured as previously described [55, 56]. Briefly, 100 μL of reaction buffer containing sodium phosphate (750 mM, pH 9.0), 0.5 mM NADP^+^, 0.5 mM α-ketoglutarate, 0.5 mM dithiothreitol (DTT), 10 μg GABase (Sigma-Aldrich, Shanghai, PRC) and 10 μL of filtered supernatant were incubated for 1 h at 37 °C, and the absorbance at 340 nm was measured using an Infinite M200 Pro plate reader (Tecan Group Ltd., Männedorf, Switzerland). The concentration of GABA was determined by comparison of the observed absorbance versus a calibration curve obtained with standard solutions of GABA analyzed in the same conditions.

### Ammonia release assay

The ammonia release assay was performed as previously described [57]. Cells grown in LBG medium acidified by HCl to initial pH 4.5 were harvested in exponential phase with OD_600_ 0.5–0.6 and in stationary phase after being cultured for 16 h, by centrifugation. The pellet was washed once with citrate buffer (25 mM, pH 4.5), pelleted again, and resuspended to 1 OD_600_/mL with 10 mL citrate buffer (25 mM, pH 4.5) supplemented with 10 mM l-glutamine. The concentration of ammonium ions was continuously measured at 25 °C using a SevenCompac™ S220 electrochemical analytical meter (Mettler-Toledo AG, Schwerzenbach, Switzerland) equipped with an ammonia ion selective electrode (model DX218-NH_4_^+^, Mettler-Toledo GmbH, Schwerzenbach, Switzerland).

### Transcriptome analysis by RNA-seq

*Escherichia coli* MG1655 and strain 3-36 were cultured overnight in LBG medium of pH 7.0. A 100 μL aliquot of the cultures was inoculated into 10 mL of LBG medium acidified by HCl to pH 4.5 and the cells were grown to exponential phase up to OD_600_ 0.5–0.6. RNA extraction and RNA-seq were carried out by CapitalBio Corporation (Beijing, China). Total RNA was extracted using the TRIzol reagent kit (Invitrogen Life Technologies, Carlsbad, CA, USA) and RNeasy MinElute cleanup kit (Qiagen, Valencia, CA, USA) following the indications of the respective manufacturers. RNA sequencing was carried out with an Illumina HiSeq 2500 system (Illumina, San Diego, CA). The gene expression difference was analyzed by Cuffdiff algorithm [58]. Genes with |log_2_(fold change)| ≥ 1 and p-value < 0.05 were assigned as differentially expressed genes. Three biological replicates of each strain were analyzed to allow for statistical analysis.

## Results

### Influence of H-NS on acid tolerance of *E. coli*

**Fig. 1:**
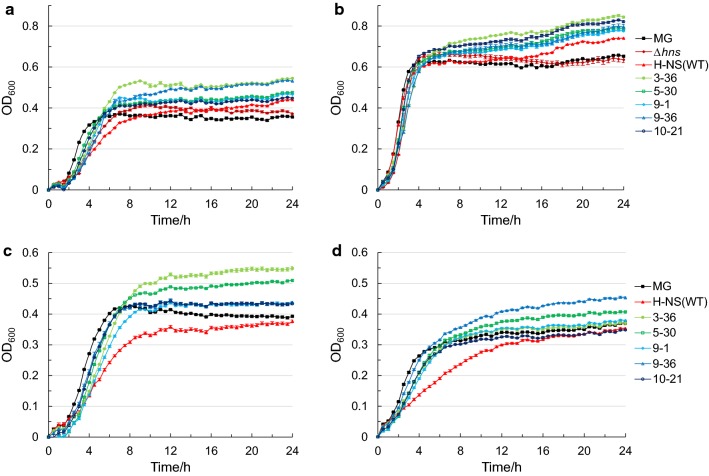
Growth of strains harboring H-NS mutants under acid stress. The change of OD_600_ of strains MG, Δ*hns*, H-NS(WT) and strains harboring H-NS mutants cultured for 24 h in LBG medium with different initial pH values obtained by the addition of HCl: **a** pH 4.5 acidified by HCl, **b** pH 7.0 (not acidified), **c** pH 5.35 acidified by acetic acid and **d** pH 4.5 acidified by succinic acid. Error bars indicate the standard error of at least three biological replicates

**Fig. 2:**
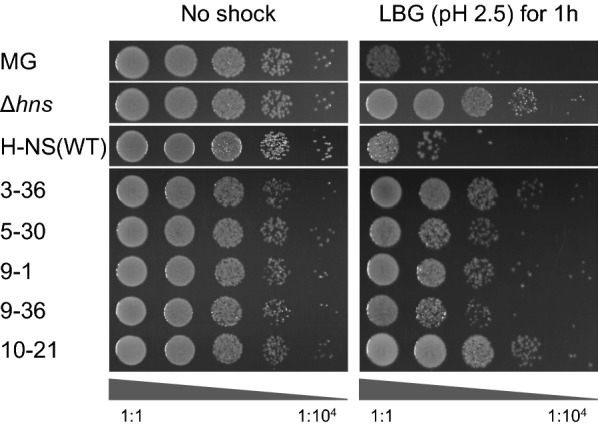
Survival of strains harboring H-NS mutants after acid shock. Comparison of the viability of strains MG, H-NS(WT) and strains harboring H-NS mutants incubated for 1 h in LBG medium (left panel) and LBG medium acidified by HCl to pH 2.5 (right panel) The images represent serial dilutions of the cultures in 10-fold steps (from left to right: 1:1 to 1:10,000)

A previous study showed that the overexpression of *hns* initiated by the strong promoter λP_L_ on the high copy number plasmid pTZ18R is lethal and incompatible with cell survival [59]. Based on this information, we chose to test the effect of H-NS on acid tolerance by using the native promoter and a low copy number plasmid. The *hns* gene containing the native promoter (1018 bp) was ligated into the low copy number plasmid pBR322 (15–20 copy numbers) [60] to construct strain H-NS(WT) or deleted from the genome via scarless CRISPR-Cas9 genome editing [51] to construct strain Δ*hns*. A growth assay under moderate acid stress (i.e., 24 h of incubation in LBG medium acidified to pH 4.5 by HCl) and an acid shock assay under extreme acid stress (i.e., 1 h of incubation in LGB medium acidified to pH 2.5 by HCl) were performed to investigate the effect of overexpression and knockout of *hns* on acid tolerance. Under moderate acid stress, only the final OD_600_ value of strain H-NS(WT) (i.e. the strain overexpressing *hns*) was 24% higher than that of the wild type strain (MG) (Fig. 1a). However, both modified strains showed growth disadvantages in exponential phase. It is worth to note that the pH value remained constant at pH 4.5 during the whole fermentation period (data not shown). Overexpression of *hns* had no effect on the survival under extreme acid stress, while the knockout of *hns* showed an increase of the survived cell number of about 100-fold, compared to the wild type strain MG (Fig. 2).

### Selection and characterization of acid tolerant mutants

After we confirmed that H-NS has a significant influence on acid tolerance and that the overexpression of *hns* can improve the growth under moderate acid stress, a library of H-NS mutants was constructed by a standard error-prone PCR protocol. The native promoter of *hns* was mutated together with the gene considering the dramatic effect of expression level on the growth [59]. The library was subjected to selection considering both growth under moderate acid stress (pH 4.5) and survival rate after acid shock (pH 2.5) as described in “Methods” section. After selection, the five mutants 3-36, 5-30, 9-1, 9-36 and 10-21 were obtained.

As shown in Fig. 1a, the final OD_600_ values of strains 3-36, 5-30, 9-1, 9-36 and 10-21 after culturing for 24 h under moderate acid stress were enhanced by 53, 33, 31, 49 and 26% compared with strain MG, respectively. The maximum growth rate of strains harboring H-NS mutants in exponential phase was higher than that of strain H-NS(WT), but lower than that of strain MG (Additional file 1: Table S1). The growth in LBG medium of pH 7.0 was also assayed (Fig. 1b). Under this condition, all of the strains harboring H-NS mutants showed a growth advantage compared with strain MG and after 24 h the OD_600_ value of the best strain, strain 3-36, was 29% higher that of strain MG.

An acid shock assay was subsequently performed to test the acid tolerance of mutants towards extreme acid stress. All the strains harboring H-NS mutants showed a higher survival rate than strain MG (Fig. 2). In particular, compared to strain MG, the number of cells that survived the acid shock was nearly 100-fold higher for mutants 3-36, 9-1, 10-21, and 10-fold higher for mutants 5-30 and 9-36. These results indicated that mutations of H-NS can result in an increase of growth under moderate acid stress and of survival rate under extreme acid stress.

We have also checked the morphology of cells harboring H-NS mutants. Cells of strain H-NS(WT) were much longer in size than those of strain MG, but the morphology of cells harboring H-NS mutants was similar to those of strain MG (Additional file 2: Figure S1).

### Growth under moderate acid stress induced by other acids commonly encountered in fermentations

The accumulation of acidic metabolites, in particular acetic acid and succinic acid, is commonly encountered during fermentations. Acetic acid is a well-known growth inhibitor found in lignocellulosic hydrolysates [2] and a typical by-product during fermentation [7]. Succinic acid is a valuable chemical useful for biorefining applications [9]. To explore whether the mutation of H-NS could confer tolerance in acid environments commonly encountered during fermentations, growth assays were performed in LBG medium acidified by the two common acidic metabolites acetic acid and succinic acid. All the strains harboring H-NS mutants showed higher OD_600_ compared with strain MG when cultured in LBG medium acidified by acetic acid to pH 5.35 (Fig. 1c). Strain 3-36, which showed the highest growth when the medium was acidified by HCl, also showed the highest OD_600_ in acetic acid environment with a 39.7% improvement over strain MG. In LBG medium acidified by succinic acid to pH 4.5, only strains 5-30 and 9-36 had a slight growth advantage (Fig. 1d), with final OD_600_ values 9.6 and 22.1% higher than that of strain MG, respectively.

### Transcriptional expression profile of H-NS mutant strain 3-36

To determine the transcriptional changes in strains harboring mutant H-NS and elucidate how the mutation of H-NS confers acid tolerance, strain 3-36, which showed the highest growth in moderate acid conditions, and strain MG were grown to exponential phase under moderate acid stress then their transcriptomes were determined and compared. The analysis showed that the mutation of H-NS changed the expression of a large number of genes (Additional file 3: Table S2). A total of 215 genes were differentially expressed with more than 2-fold change and p-value < 0.05 between strain 3-36 and strain MG, including 128 genes up-regulated and 87 genes down-regulated. Among them, only 24 genes have already been reported to be directly regulated by H-NS.

**Fig. 3:**
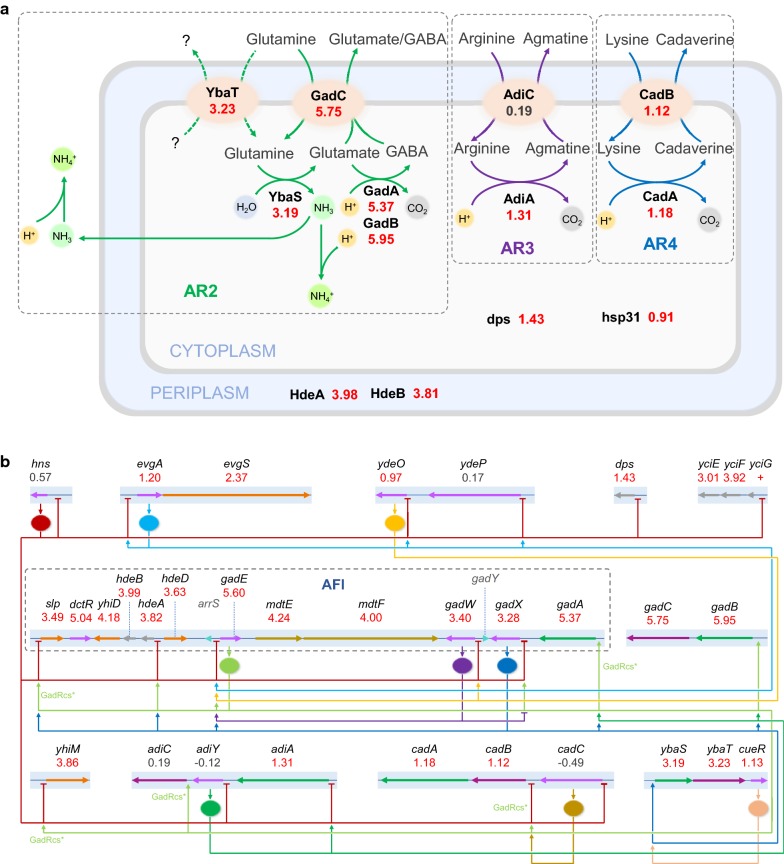
Transcriptome analysis of strain 3-36 compared with strain MG. **a** Differentially regulated genes involved in acid resistance mechanisms. **b** Influence of 3-36 on the regulatory network of acid tolerance. The number below each gene is the log_2_(fold change) of strain 3-36 compared with strain MG. Red values: up-regulated in 3-36. *AFI* acid fitness island

Noteworthy, many genes involved in the acid tolerance were highly up-regulated in strain 3-36 (Fig. 3a). The transcription of *ybaS*, *ybaT*, *gadA*, *gadB* and *gadC*, which compose the AR2 system, increased 9.1-, 9.4-, 41.4-, 61.8-, and 53.8-fold, respectively. This is consistent with the results obtained with the GAD activity assay and the GABA and ammonia release assays (see below). Similarly, the transcription of *adiA*, *cadA* and *cadB*, which belong to the AR3 and AR4 systems, increased 2.5-, 2.3-, 2.2-fold, respectively. Moreover, *hdeA* and *hdeB*, which express periplasmic chaperones and *dps* and *hsp31*, which express cytoplasmic chaperones, were upregulated 14.1-, 15.9-, 2.7-, 1.9-fold, respectively. The transcription of *yciE* and *yciF* in *yciGFE* operon was also highly up-regulated (8.1- and 15.1-fold, respectively) (Fig. 3b). The gene *yciG* is considered an important regulator for the tolerance to acid stress, oxidative stress and thermal stress in stationary phase [61]. *yciE* and *yciF* are located in the same operon expressing heat shock proteins. Additionally, the transcription of *otsA* and *otsB*, which convert UDP-glucose to trehalose, a disaccharide believed to protect the cells against various physical and chemical stresses [62], increased 3.3- and 3.9-fold (Additional file 3: Table S2).

The H-NS mutant also altered the expression of transcriptional regulators. Among them, the transcription of *evgA*, *evgS*, *ydeO*, *gadE*, *gadW*, *gadX* and *cueR*, which are crucial in acid tolerance [41], increased 2.3-, 5.2-, 2.0-, 48.5-, 10.6-, 9.7- and 2.2-fold, respectively (Fig. 3b). These results suggested that mutations of H-NS improve the acid tolerance of *E. coli* by activating multiple acid resistance systems through the regulatory network.

### Activation of the AR2 system

**Fig. 4:**
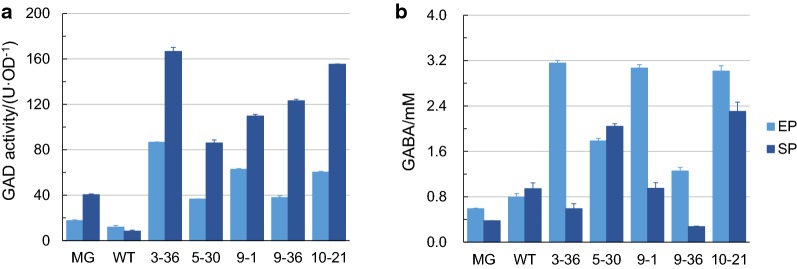
Activation of the AR2 system by H-NS mutants: GAD activity and GABA release. **a** GAD activity of cells harboring wild type H-NS and H-NS mutants in exponential phase (EP) and stationary phase (SP). **b** Concentration of GABA released by cells harboring wild type H-NS and H-NS mutants in EP and SP after 1 h incubation with excess of l-glutamate in citrate buffer (25 mM, pH 4.5). Error bars indicate the standard error of at least two biological replicates

Acid tolerance is a complex trait regulated by several acid resistance systems (ARs) [26]. Among the known ARs, the AR2 system, which acts by consuming intracellular protons, is considered to be the most important under fermentative conditions [63]. The AR2 system is originally reported as glutamate-dependent and consists of the glutamate decarboxylases GadA and GadB and the glutamate/GABA antiporter GadC [48]. To elucidate how the AR2 system was implicated in the mechanism behind the observed acid tolerance, the GAD activity of H-NS mutant strains cultured up to both exponential phase and stationary phase were assayed. The GAD activity of all the mutant strains was higher than that of the MG strain in both exponential phase and stationary phase. In particular, the GAD activity of strains 3-36, 5-30, 9-1, 9-36 and 10-21 was 4.9-, 2.1-, 3.6-, 2.2- and 3.4-fold higher than that of strain MG in exponential phase, and 4.1-, 2.1-, 2.7-, 3.0- and 3.8-fold higher than that of strain MG in stationary phase, respectively (Fig. 4a).

GABA would be released to the medium during the process of AR2 functioning [64]. The amount of GABA released by the cells in exponential phase and stationary phase was measured to further explore the influence of AR2 in the acid tolerance displayed by H-NS mutants. As observed for the GAD activity, the release of GABA of all the mutant strains was higher than that of the MG strain in both exponential phase and stationary phase. In particular, the amount of GABA released by strains 3-36, 5-30, 9-1, 9-36 and 10-21 was 5.4-, 3.0-, 5.2-, 2.1- and 5.1-fold higher than that released by strain MG in exponential phase, and 1.6-, 5.4-, 2.5-, 0.7- and 6.1-fold than that released by strain MG in stationary phase, respectively (Fig. 4b).

**Fig. 5:**
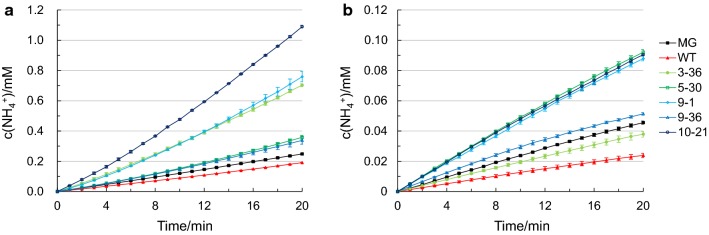
Enzymatic release of ammonia of cells harboring H-NS mutants. Accumulation of ammonium ions obtained by incubating cells harboring wild type H-NS and H-NS mutants in citrate buffer (25 mM, pH 4.5) containing l-glutamine in excess: **a** cells in exponential phase (EP) and **b** cells in stationary phase (SP). Error bars indicate the standard error of at least two biological replicates

Furthermore, the involvement of glutamine in the mechanism of acid tolerance operated by the AR2 system was recently reported [57]. According to this model, glutamine is imported into the cytoplasm by GadC [57] or YbaT [65], and converted into glutamate and ammonia by YbaS. The released ammonia is then protonated into ammonium ions while neutralizing the acids present in the cytoplasm. Therefore, the level of ammonium ions released by the cells in citrate buffer of pH 4.5 with l-glutamine in excess was monitored to further test the activation of the AR2 system in H-NS mutants. All strains harboring H-NS mutants had a higher rate of ammonium release except strain 3-36 in stationary phase (Fig. 5). In particular, the rate of ammonium release of strains 3-36, 5-30, 9-1, 9-36 and 10-21 in exponential phase was 2.8-, 1.4-, 3.1-, 1.4- and 4.4-fold higher than that of strain MG, respectively (Fig. 5a**)**. The rate of ammonium release of strains 3-36, 5-30, 9-1, 9-36 and 10-21 in stationary phase was 0.8-, 2.0-, 1.9-, 1.1- and 2.0-fold of strain MG (Fig. 5b), respectively. Taken together, these results demonstrated the involvement of the AR2 system in the acid tolerance showed by the H-NS mutants.

### Sequence alignment and mutation analysis of the mutants

**Fig. 6:**
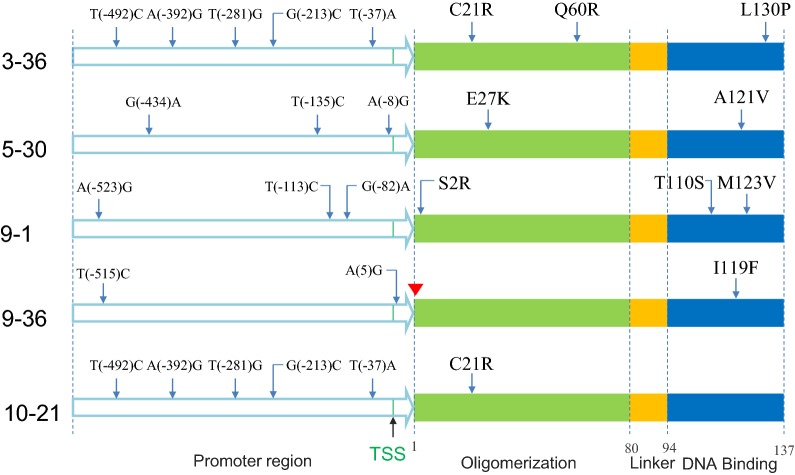
Summary of the mutations in the promoter region and the primary sequence of H-NS mutants. The red triangle indicates that the start codon was mutated from the native AUG to ACG without amino acid mutation [76]

The full-length H-NS structure was previously determined by solid-state NMR [66]. H-NS is comprised of two functional domains, a N-terminal oligomerization domain (residues 1-79) and a C-terminal DNA binding domain (residues 95-137), separated by a flexible linker (residues 80-94) [67]. The amino acid mutations together with the mutations in the promoter region of the H-NS mutants were sequenced and aligned and are summarized in Fig. 6. Interestingly, all the mutants had mutations in the promoter region and coded region, a total of nine mutations (S2R, C21R, E27K, Q60R, T110S, I119F, A122V, M123V, L130P) were found. Among them, four mutations (S2R, C21R, E27K, Q60R) fell into the N-terminal oligomerization domain, while five mutations (T110S, I119F, A122V, M123V, L130P) were located in the C-terminal DNA binding domain. Given the number of mutations in the promoter region, qTR-PCR was further carried out to determine the transcription levels of these mutants, and it was found these mutations all increased the transcription by various degrees compared with strain MG (Additional file 4: Figure S2).

## Discussion

Acid stress arising from biomass hydrolysates and acidic metabolic products can damage cells and impair their growth and fermentation yield [9, 10, 68, 69]. Previous studies based on global transcription machinery engineering (gTME) employed tailored transcriptional regulators acting as activators to improve the tolerance of *E. coli* strains to environmental stress [14, 21, 30, 70, 71]. The possibility to use regulators acting as repressors to achieve the same goals has not yet been investigated. In this work, we investigated the influence of the global regulator and repressor H-NS on acid tolerance and we engineered H-NS to improve the acid tolerance of the commonly used workhorse *E. coli*. The five mutants obtained showed growth advantages in moderate acid environment and a higher survival rate in extreme acid environment compared to the wild type strain MG. Strain 3-36 harboring the best H-NS mutant in this study grew slightly better than a previously reported strain AcM1 harboring CRP mutant (53% versus 46%) under moderate acid stress [15].

The comparative transcriptome analysis showed that H-NS mutations can up-regulate several genes involved in well-studied acid resistance mechanisms including protective chaperones, and the proton-consuming AR2, AR3 and AR4 systems [26]. The results of GAD activity assay and ammonia and GABA release assays confirmed the up-regulation of the AR2 system in these H-NS mutants. The transcription of *sodC* and *katE* was also increased (2.5- and 2.8-fold, respectively) (Additional file 3: Table S2). *SodC* and *katE*, which express superoxide dismutase and catalase II, respectively, are involved in removing intracellular ROS (reactive oxygen species) which often increase significantly when cells are under stress conditions [21, 72, 73]. The up-regulation of these two genes in our H-NS mutants, together with the up-regulation of *katE* previously observed in another acid-tolerant strain [15], suggests that the removal of ROS is an important aspect of acid tolerance. Taken together, these findings confirms the crucial role of H-NS in the regulation of acid tolerance [41].

In addition to the tolerance showed towards HCl, the strains harboring H-NS mutants exhibited tolerance also towards acetic acid and succinic acid, although the performance depended on the type of acid used to induce the stress (Fig. 1). For instance, strain 3-36 showed the best growth performance in media acidified by HCl (pH 4.5) and acetic acid (pH 5.35) but it showed only a slight growth advantage in medium acidified by succinic acid (pH 4.5). This result was not surprising as a similar behavior was also observed in previous studies [15, 74]. Such dependence likely results from two factors: the different amount of acid that remains undissociated in the medium, which influences its passive transport across biological membranes, and the different toxicity of the conjugate bases [9].

Sequencing results provided some useful insights into the mechanism behind the acid tolerance of the H-NS mutants. H-NS acts almost exclusively as a repressor [75], and it is estimated to have approximately 20,000 copies per genome [75]. H-NS binds to about 250 loci, mostly in fimbriae- and phage-related genes and IS (insertion sequence element)-inserted genes, with a particular preference to horizontally acquired DNA [33]. H-NS directly represses more than 20 genes involved in acid tolerance and almost half of them are transcriptional regulators, including *evgA*, *ydeO*, *gadE*, *gadW*, *gadX*, *adiY*, *cadC* [41]. All the H-NS mutants of this study had mutations within the promoter region, which resulted in a modest increase of the transcription levels of H-NS, except for the mutant 9-36, whose transcription was enhanced by 16.8-fold (Fig. 6 and Additional file 4: Figure S2). This enhanced transcription, however, is unlikely to be reflected in an increased translation because the start codon for this mutant was changed from the native AUG to ACG, which can sharply decrease the translation initiation efficiency [76]. Indeed, as can be seen in Additional file 5: Figure S3, the expression level of each H-NS mutant, as determined by a GFP assay, was comparable to that of wild-type (50–89%), except for the mutant 9-36. For this mutant, as we suspected, the expression level was only 3–13% of the wild type. Among the nine mutations in the coded region of the H-NS mutants, five mutations (T110S, I119F, A122V, M123V and L130P) were located in the DNA binding region, and one of them (T110S) in the highly conserved core of the DNA binding motif [75]. It has been reported that several mutations at sites T110, I119, A122, M123 and L130 (DNA binding region) reduce the binding of H-NS to DNA or are involved in loss of function for H-NS [77, 78], while the mutation at site C21 (oligomerization region) increases the susceptibility of H-NS to Lon protease [42, 75]. Based on this information, we surmise that the H-NS mutants found in this study must be defective in DNA binding and since H-NS functions in a tetrameric form, the defective H-NS monomers might act as a spoiler in the formation of functional H-NS oligomers, thereby reducing the repression of H-NS. This mechanism would be consistent with the growth disadvantage that was observed in the exponential phase for the strain carrying the wild type H-NS on the plasmid (Fig. 1a). It cannot be excluded that these mutations may also induce more subtle changes to the binding of H-NS to other loci. While further investigation is required to fully understand the mechanism behind the acid tolerance of H-NS mutants, this work does suggest that fine-tuning the repressive action of H-NS can be a useful approach for improving acid tolerance in *E. coli*.

## Conclusions

The influence of the global regulator H-NS on the acid tolerance of *E. coli* was studied. We showed that the H-NS mutants improved the acid tolerance by activating multiple acid resistance mechanisms, especially the acid resistance system AR2. This study proved that the engineering of global regulators that act as repressors can be a successful strategy to obtain strains with phenotypes of interest, thereby expanding the spectrum of application of gTME.

## Additional files

**Additional file 1: Table S1.** Maximum growth rate of strains harboring H-NS mutants under acid stress.

**Additional file 2: Figure S1.** Cell morphology of strains MG, and strains harboring H-NS wild type or mutants.

**Additional file 3: Table S2.** Genes differently expressed at transcriptional level between strain 3-36 and wild type strain MG.

**Additional file 4: Figure S2.** Fold change of the expression of H-NS at transcriptional level obtained by qRT-PCR.

**Additional file 5: Figure S3.** Quantification of protein expression level of H-NS wild type and mutants in exponential phase (EP) and stationary phase (SP). Green fluorescent protein (GFP) was fused to the C-terminal of H-NS wild type and mutants. Cells expressing the fusion protein were cultured to exponential phase or stationary phase in LBG medium acidified by HCl to pH 4.5. Fluorescence was determined to quantify the protein expression level.

## Abbreviations

OD_600_: optical density at 600 nm
gTME: global transcription machinery engineering
AR2: acid resistance system 2
AR3: acid resistance system 3
AR4: acid resistance system 4
ORF: open reading frame
GAD: glutamate decarboxylase
GABA: γ-amino butyric acid
EP: exponential phase
SP: stationary phase
AFI: acid fitness island
