# Supplementary material for: Engineered global regulator H-NS improves the acid tolerance of E. coli
Source: Microb Cell Fact. 2018 Jul 27;17:118. doi: 10.1186/s12934-018-0966-z (PMC6064147; doi:10.1186/s12934-018-0966-z)
Supplement: Supplementary file 1 — Additional file 1: Table S1. Maximum growth rate of strains harboring H-NS mutants under acid stress. [file 12934_2018_966_MOESM1_ESM.docx]

**Table S1** Maximum growth rate of strains harboring H-NS mutants under acid stress ^a^.

| Maximum growth rate /(OD•h^-1^) | LBG medium of initial pH 4.5 acidified by HCl | LBG medium of initial pH 7.0 | LBG medium of initial pH 5.35 acidified by acetic acid | LBG medium of initial pH 4.5 acidified by succinic acid |
| --- | --- | --- | --- | --- |
| MG | 0.1503 | 0.3130 | 0.1193 | 0.0953 |
| *Δhns* | 0.1187 | 0.3033 | - ^b^ | - |
| WT | 0.0640 | 0.2723 | 0.0570 | 0.0313 |
| 3-36 | 0.1187 | 0.2040 | 0.0917 | 0.0640 |
| 5-30 | 0.1297 | 0.2930 | 0.1073 | 0.0743 |
| 9-1 | 0.1187 | 0.2623 | 0.0823 | 0.0627 |
| 9-36 | 0.1240 | 0.2283 | 0.1050 | 0.0830 |
| 10-21 | 0.1260 | 0.3053 | 0.1053 | 0.0713 |

a. Calculated from Fig. 1.

b. No data.
