# Supplementary figures and images for: Engineered global regulator H-NS improves the acid tolerance of E. coli
Source: Microb Cell Fact. 2018 Jul 27;17:118. doi: 10.1186/s12934-018-0966-z (PMC6064147; doi:10.1186/s12934-018-0966-z)

EP

SP

MG

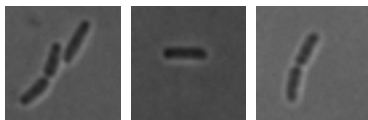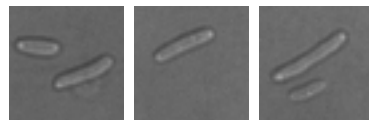

H-NS(WT)

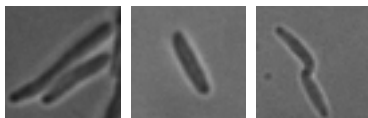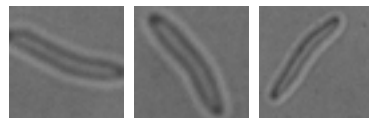

3-36

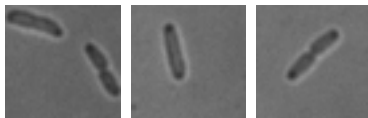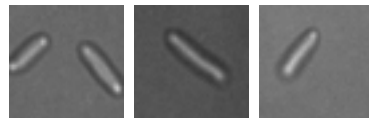

5-30

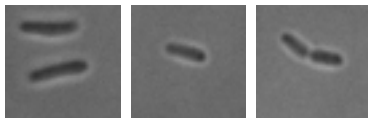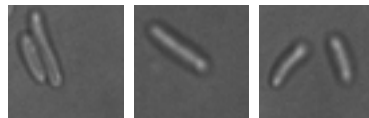

9-1

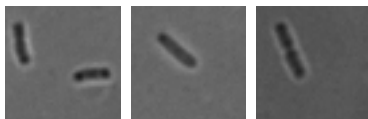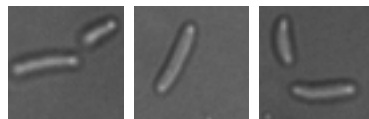

9-36

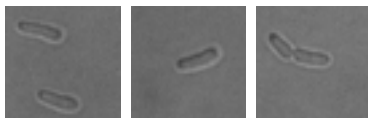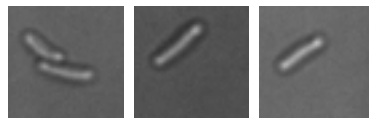

10-21

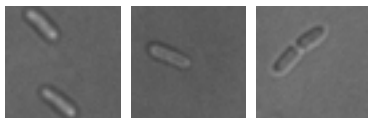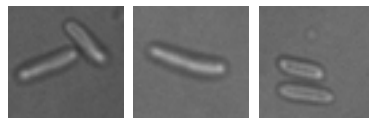

Supplement: Supplementary file 2 — Additional file 2: Figure S1. Cell morphology of strains MG, and strains harboring H-NS wild type or mutants. [file 12934_2018_966_MOESM2_ESM.pdf]

**Fold Change**

20.0  
16.0  
12.0  
8.0  
4.0  
0.0

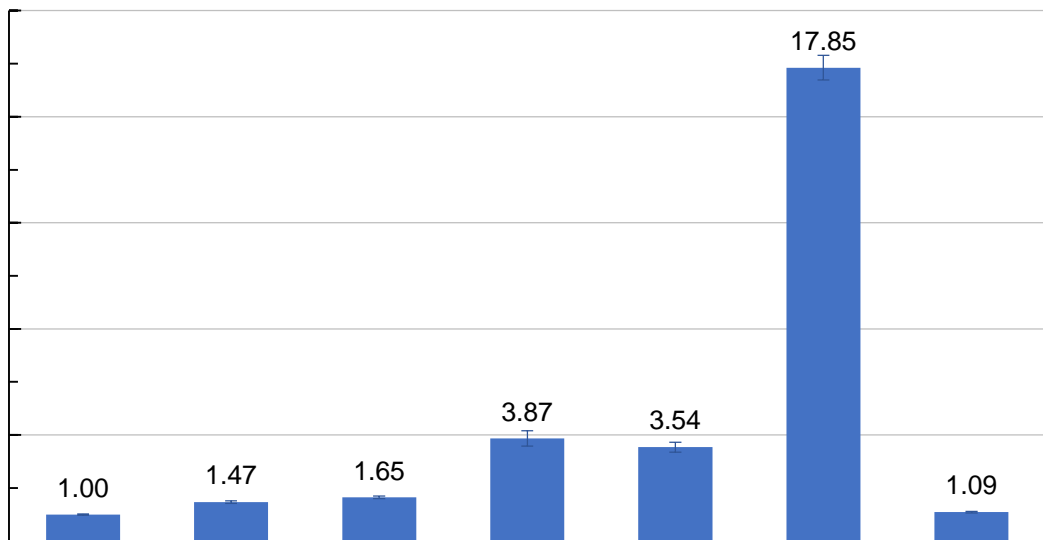

MG

H-NS(WT)

3-36

5-30

9-1

9-36

10-21

Supplement: Supplementary file 4 — Additional file 4: Figure S2. Fold change of the expression of H-NS at transcriptional level obtained by qRT-PCR. [file 12934_2018_966_MOESM4_ESM.pdf]

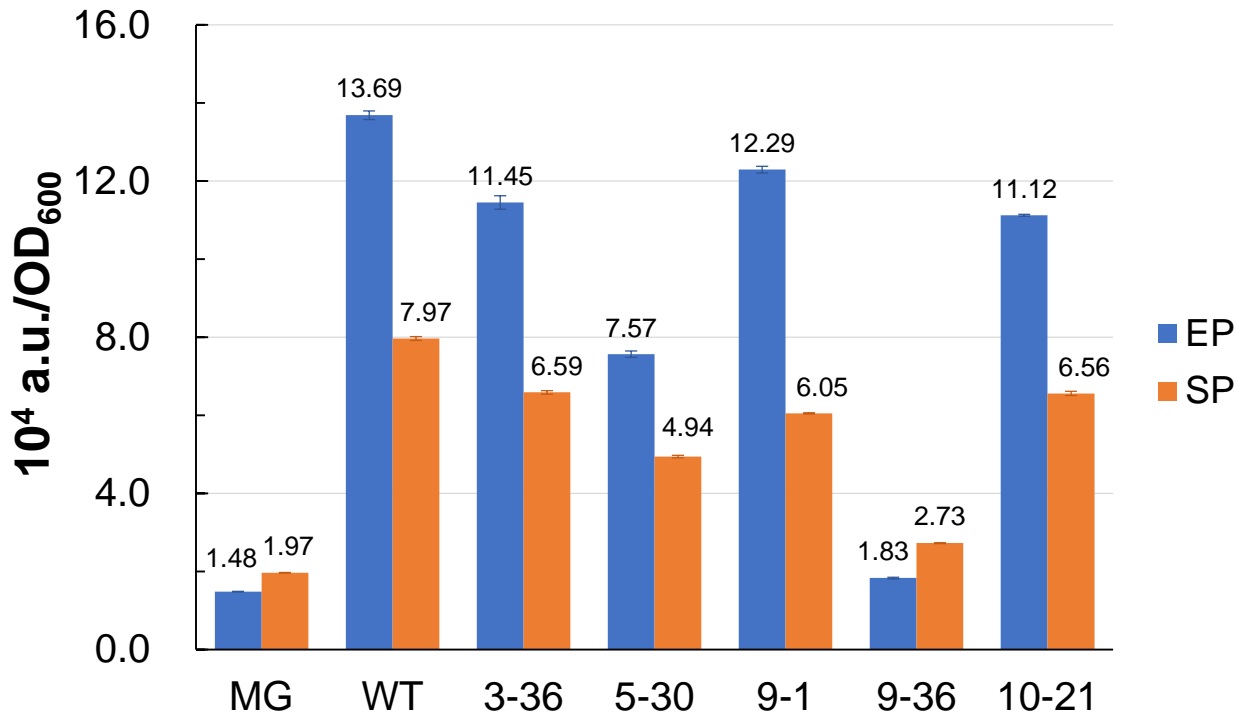

Supplement: Supplementary file 5 — Additional file 5: Figure S3. Quantification of protein expression level of H-NS wild type and mutants in exponential phase (EP) and stationary phase (SP). Green fluorescent protein (GFP) was fused to the C-terminal of H-NS wild type and mutants. Cells expressing the fusion protein were cultured to exponential phase or stationary phase in LBG medium acidified by HCl to pH 4.5. Fluorescence was determined to quantify the protein expression level. [file 12934_2018_966_MOESM5_ESM.pdf]
